# What is the long term impact of voucher scheme on primary care? Findings from a repeated cross sectional study using propensity score matching

**DOI:** 10.1186/s12913-019-4707-8

**Published:** 2019-11-21

**Authors:** Carrie H. K. Yam, Eliza L. Y. Wong, Valerie L. H. Fung, Sian M. Griffiths, Eng-Kiong Yeoh

**Affiliations:** The Jockey Club School of Public Health and Primary Care, The Chinese University of Hong Kong, Hong Kong, SAR People’s Republic of China

**Keywords:** Voucher, Preventive care, Disease prevention, Chronic disease

## Abstract

**Background:**

Vouchers are increasingly used as a demand-side subsidy to reduce financial hardship and improve quality of services. Elderly Healthcare Voucher Scheme has been introduced by the Hong Kong Government since 2009 to provide subsidy to elderly aged 65 and above to visit ten different types of private primary care providers for curative, preventive and chronic disease management. Several enhancements have been made over the past few years. This paper (as part of an evaluation study of this unique healthcare voucher scheme) aims to assess the long term impact of the voucher scheme in encouraging the use of primary care services.

**Methods:**

Two rounds of cross-sectional survey among elderly in Hong Kong were conducted in 2010 and 2016. Propensity score matching and analysis were used to compare changes in perception and usage of vouchers over time.

**Results:**

61.5% of respondents in 2016 agreed “the scheme encourages me to use more private primary care services”, a significant increase from 36.2% in 2010. Among those who agreed in 2016, the majority thought the voucher scheme would encourage them to use acute services (90.3%) in the private sector, rather than preventive care (40.3%) and chronic disease management (12.2%). Respondents also reported that their current usual choice of care was visiting “both public and private doctors” (61.9%), representing a significant increase (up from 48.4%) prior to their use of voucher.

**Conclusions:**

The voucher scheme has encouraged the use of more private care services, particularly acute services rather than disease prevention or management of chronic disease. However, there needs to be caution that the untargeted and open-ended nature of voucher scheme could result in supply-induced demand which would affect long term financial sustainability. The dual utilization of health services in both the public and private sector may also compromise continuity and quality of care. The design of the voucher needs to be more specific, targeting prevention and chronic disease management rather than unspecified care which is mainly acute and episodic in order to maximize service delivery capacity as a whole for equitable access in universal health coverage and to contribute to a sustainable financing system.

## Background

### Primary care and private sector role

Aspiration to provide better primary care is the basis of healthcare reform agendas in many countries around the world to deliver better health outcomes and improve efficiency and quality of care [1] and Hong Kong is no exception [2]. The challenge for healthcare systems is to shift from a focus on acute episodic illness and hospital centric systems to a primary care and community based system to meet the needs of the ageing population. Not only is the elderly population growing, the globalization of unhealthy lifestyles has resulted in the rise of long-term chronic conditions and preventable illnesses requiring multiple complex health and social care interventions over many years [3]. This places greater pressure on both primary and secondary care services [4, 5]. New models of primary care services - defined as first access care - are thus increasingly important [6]. In order to address the challenges, health systems around the world are increasingly orienting resources towards primary care and a community-based model of chronic care placing emphasis on better integration of services at both vertical and horizontal levels to improve the continuity and quality of care for frail elderly persons and patients with chronic diseases and to reduce the need for hospital admission [7]. Multiple interventions are needed, including those that target organizational and behavioral changes. Self-management and lifestyle interventions can be supported in primary care community setting which also coordinate the transitions of chronic care with secondary care and long term care. Interventions that change resources, facilities or processes are also necessary in shifting towards more outpatient and ambulatory services through investment in home care, nursing homes and hospices; repurposing hospitals for acute complex care only; and ambulatory facilities for day surgery, chronic facility-based care, and day hospital.

Governments are increasingly recognizing the growing role of the private sector in both financing and providing inpatient, outpatient and ambulatory care [8]. Globally, engaging and interacting with the private sector in initiatives, collectively referred to as public-private partnerships (PPPs), is growing in order to leverage private capacity and promote greater participation from the private sector in health services through different forms of collaboration to enhance the capacity of health systems and services quality [9]. Through creating the public-private partnerships, it aims to create equity by increasing affordability and accessibility of private services among the general population via providing public subsidy or purchasing private services, lessen the burden of the public healthcare sector by reallocating demand in public services to the private market, and improve quality of services through introducing competition. Deber suggests that a range of policy instruments are available to actors or agencies in different health systems to influence private sector engagement in contributing to health system goals such as: exhortation through dissemination of information; taxation which manipulates incentives and disincentives; subsidization of expenditures; regulation through setting rules of behaviors; and public ownership [10]. Vouchers are a form of demand-side subsidy used to encourage behavioral changes with respect to under-utilized services such as preventive care [11–13]. They have been widely used as a tool to increase the utilization of under-utilized services. Since early 2000, vouchers have been targeted at vulnerable groups to reduce barriers to access in developing countries [14]. For example, vouchers are used for mosquito bed nets for malaria control in Tanzania [15], treatment of sexually transmitted infection for sex workers in Nicaragua [16], and maternal and family planning in Bangladesh [17, 18]. A systematic review of healthcare voucher schemes has demonstrated evidence that health voucher programs have been successful in increasing utilization of health services which are well-defined and time-limited, in particular those services which are under-utilized [19, 20].

Experience of vouchers for healthcare in developed economies is very limited. Another and more critical observation is healthcare vouchers have not been applied as a purchasing mechanism for an entire population group for an unspecified range of primary care services. This study is part of an evaluation of the effectiveness and impact of a unique healthcare voucher program implemented in Hong Kong Special Administrative Region (SAR) of China which is a developed economy. The Elderly Healthcare Voucher Scheme provides financial subsidies to the entire Hong Kong resident population aged 65 and above for unspecified healthcare services provided by 10 categories of primary care practitioners.

### Primary healthcare in Hong Kong

Hong Kong’s health system is segmented and compartmentalized in financing and delivery. It has parallel systems of public and private financing for healthcare. Public financing is from general tax revenue and accounts for 51% of total health expenditure in 2015/16 [21]. The equidominant private system accounts for 49% of total health expenditure, and is mainly financed by household out-of-pockets (70%) with 29% financed through privately purchased or employer-based insurance [21]. Public policy undertakes to provide universal health coverage to the entire resident population. The public sector provides 90% of services in hospital care, but only 30% of primary healthcare [22]. Primary care in Hong Kong is largely provided in the private sector. The private sector provides 70% of primary outpatient care, but only 10% of inpatient hospital care [22]. The services fee of public general outpatient (primary care) clinics is HK$50 per attendance while that of private sector is at higher fees ranging from HK$300 for primary consultation to HK$800 for specialist practice [23]. Public general outpatient clinics are primarily used by the elderly, low-income groups and chronically ill patients. In 2018, elderly patients aged 65 or above accounted for one-third of patients in the public general out-patient clinics [24]. Willingness for pay for private primary care services by elderly persons is low because they perceived the services as unaffordable and they have concerns on the service quality [25]. Despite long waits and crowded conditions, many elderly persons tended to prefer public over private primary care services because of lower clinic fees and relatively higher trust in public services [26–28]. The segmented public-private financing and delivery system has been put under increasing pressure to meet the demands and needs of an ageing population for equity in access particularly for primary care. Hong Kong has one of the higher life expectancy - 82 years for mean and 88 years of women [29]. 17% of the population are aged 65 and above, and 75% of this population group has at least one chronic diseases and 15% have more than one chronic diseases [22].

### Elderly healthcare voucher scheme in Hong Kong

**Fig. 1:**
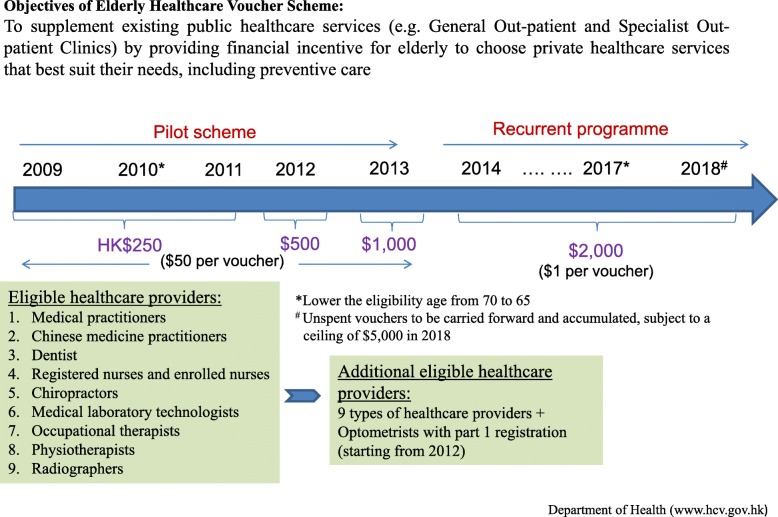
Design of Voucher scheme

Access to public primary care is an issue in view of the under-provision. Affordability remains a significant barrier to effective health system utilization, especially for the elderly persons particularly those with chronic diseases who need to make high amount of out-of-pocket consultation payment in the private sector. To reduce reliance on public healthcare services and to relieve the financial burden of elderly, the Government of Hong Kong SAR introduced the Elderly Health Care Voucher Scheme in 2009 for the entire Hong Kong resident population aged 70 and above. It is intended to supplement existing public healthcare services by providing a financial incentive for elderly to choose private healthcare services that best suit their needs including preventive care. Currently all Hong Kong residents aged 65 and above can use the vouchers to seek care from ten different types of primary healthcare professionals (including medical practitioners, Chinese medicine practitioners, dentists, chiropractors, registered nurses and enrolled nurses, physiotherapists, occupational therapists, radiographers, medical laboratory technologists and optometrists) in the private sector for curative care, preventive care and chronic disease management. The use of vouchers for inpatient services and sole purchase of health products is not allowed under the voucher scheme [30]. Using the voucher as a financial lever, the scheme was intended to stimulate elderly persons to seek more care from the community-based private sector and to promote the family doctor concept. The voucher is in electronic form. All Hong Kong residents aged 65 and over are automatically registered for the voucher scheme. Enrollment is activated when the elderly person seeks care from one of the participating primary care practitioners on presentation of their Hong Kong Identity Care (ID) and registration of their unique ID number. Initially, the voucher scheme provided five Health Care Vouchers, each worth HK$50, to all elderly people aged 70 or above annually during the first three years. A review of the pilot program conducted in 2012 in Hong Kong showed that the voucher scheme alone was not effective in encouraging the use of private primary care services from either consumer or provider perspective [26]. Public care offered by the Hong Kong Hospital Authority remained the first choice for most elderly people seeking care, especially for their chronic conditions owing to the low cost of public services and the insufficient amount of subsidy for private services offered in the voucher scheme at that time. There was also a low willingness to pay for private services among the elderly people in Hong Kong for management of chronic diseases and health checks [25]. Subsequently, the Government enhanced the voucher scheme by increasing the service providers (inclusion of optometrist in 2012), increasing the subsidy amount (increase progressively from HK$250 in 2009 to the present level of HK$2000 annually), and lowering the eligible age from 70 to 65 so as to relieve the financial burden of elderly persons (Fig. 1). Currently, the voucher amount is HK$2000 per year with a face-value of each voucher at $1 instead of the original $50 each. It was intended to provide greater flexibility in the services that can be provided and the charges that can be made. The elderly can use the electronic vouchers to partially or fully pay the services charges by the participating primary care practitioners. Co-payment is not a feature of the design and is not required as long as the amount of unspent voucher amount can meet the charges. Effectively the voucher is for the entire sum of HK$2000 and can be used for as many primary care consultations and at whatever charges that practitioner makes which the elderly persons have agreed with and within the unspent voucher amount.

With these enhancements, the proportion of eligible elderly people ever using vouchers has increased progressively from 28% in 2009 to 94% in 2018, with a corresponding increase in the annual amount of vouchers claimed from HK$40 million to HK$2.8 billion [31, 32]. The number of primary care visits through voucher scheme (in term of average number of voucher claim transactions per each voucher user) has doubled from 1.84 times in 2009 to 3.65 times in 2017 [27, 31]. The participation rate of primary care practitioners also increased from 14% in 2014 to 20% in 2017 [32]. The participation rate of optometrist was the highest at 67% in 2016, followed by dentists (44%), medical practitioners (42%) and chiropractors (36%) and Chinese medicine practitioners (32%) [31].

Developing primary care and promoting PPP are key elements of the policy agenda in Hong Kong. Hong Kong shares the health system characteristics of many other Asian health systems, considering the global need to identify innovative ways to build a sustainable health financing system, this study aims to provide important insight and greater understanding of drivers for use of the voucher scheme to achieve the objective of encouraging greater use of private primary care services and effectively managing chronic conditions amongst the elderly population in the community.

The study objectives were to assess changes over time in attitudes towards, and usage of, vouchers amongst elderly people in the community, and to assess the long term impact of the voucher scheme in encouraging the use of private primary care services. The evidence generated from this study will inform other economies whether and how demand-side financing through voucher scheme could enable the engagement of private sector for health system goals of universal health coverage.

## Methods

### Sampling

This was a repeated cross-sectional survey of elderly people aged 70 or above who are eligible for the voucher scheme. A questionnaire survey among the elderly on their perceptions towards the voucher scheme was conducted in 2010 (one year after the implementation of the initial pilot). Building on this piece of work [26], we conducted another round of cross-sectional survey among all eligible voucher users using a similar methodology to the 2010 survey. Due to the lack of a population register from which the elderly can be randomly sampled in Hong Kong, the participants were recruited through convenience sampling from three types of enumeration sites to broaden the types of elderly recruited so as to cover elderly persons with and without illnesses and from both public and private providers. The enumeration sites included (i) primary care (outpatient) clinics in both the public and private sectors for doctor consultation, and (ii) neighborhood elderly centers run by the Social Welfare Department to provide community services at neighborhood level to enable elderly to remain in the community, and (iii) elderly health centers run by the Department of Health for health assessment. The sites were chosen based on the distribution of household income in Hong Kong Island, Kowloon, and New Territories. Within each site, the eligible elderly persons were mainly referred by the clinics/ centres staff with the consent of the elderly persons. A total of 942 respondents were targeted in order to detect a change of percentage of voucher users at a marginal error of plus or minus 5% at 95% confidence level with 90% power.

### Survey data collection

The repeated cross-sectional survey was conducted face-to-face by trained interviewers with a structured questionnaire in Chinese from June 2016 to January 2017. The questionnaire consisted of four key sections including (i) demographic characteristics and healthcare services utilization pattern, (ii) awareness and understanding of the voucher scheme, (iii) vouchers usage, e.g. whether they have used vouchers for private primary care services, especially in disease prevention and chronic disease management, and reasons for not using it, and (iv) attitudes towards the voucher design and the perceived impact of voucher on their health seeking behaviors. The same set of questions relating to the design and impact were used in both the 2010 and current survey for comparison (Additional file 1). A pilot survey of 10 samples was conducted at an outpatient clinic to test the survey logistics and wording of the questions.

Ethical approval was obtained from the Ethics Committee in the Hong Kong Hospital Authority and Department of Health.

### Statistical analysis

Data was entered in a SPSS 20.0 database using dual data entry, and over 10% of the database was further audited to ensure accuracy and completeness. Each participant was assigned a unique study identifier number to ensure confidentially.

To minimize bias between the two samples in 2010 and 2016, propensity score matching was first conducted to create a matching population based on demographics and the socio-economic data of the elderly. We used logistic regression model with independent variables including gender, age, living district, living status i.e. living alone or living with others, the presence of social security assistance, and health insurance coverage to calculate the propensity score for each participant, i.e. predicted probabilities. Participants in 2010 and 2016 were then matched one-by-one based on propensity score using nearest neighbor matching. The suitability of the matching was examined by checking the characteristics of the samples in 2010 and 2016 by a Chi-square test. After matching, if there was insignificant difference between two years on the elderly’s characteristics, it indicated that selection biases were minimized. The quality of matching was also examined by the balancing score including the standardized difference of the propensity score means and the variance ratio of the propensity score [33].

Using the propensity score matching, we used Chi-square test to compare the attitude, perception and usage of vouchers across two independent cross-sectional samples in 2010 and 2016. The primary outcome was the perceived change about whether the voucher scheme can encourage greater use of private primary care services. We also conducted univariate analyses on the pooled cross-sectional data to determine associations of the perceived change on behavior with other variables adjusted by year and enumeration sites. All independent variables with *p* < 0.05 in the univariate analyses were considered for entry into the multivariate logistic regression model to identify factors associated with perceived behavior change, and to estimate adjusted odds ratio (OR) with 95% confidence intervals (CI).

## Results

### Respondents’ profile in 2016

In total, 974 elderly were successfully interviewed in 2016 (versus 1026 elderly in 2010), constituting a response rate of 78.6%. The age distribution of the elderly respondents in 2016 was comparable to that of the elderly population in Hong Kong. Overall, 34.2% of respondents were male in 2016, and about half (57.2%) were under 80 years old. Around 11.4% were recipients of comprehensive social security assistance (comparable to 9.9% of the elderly population aged 65 or above in Hong Kong) [34].

Table 1 shows the socio-economic characteristics of elderly by year before and after propensity score matching. Before matching, there was a significantly higher proportion of males and those elderly living in New Territories District in the 2010 sample, compared to 2016 sample. After propensity score matching, there was no significant difference across these two variables between 2010 and 2016. In addition, the standardized difference of the propensity score means for the two samples was 0.092 (close to zero), and the variance ratio of the propensity score between the two samples was 1.106 (close to one), indicating a good quality matches.

**Table 1:** Characteristics of respondents by year before and after propensity score (PS) matching

Covariate	Before PS match			After PS match		
	2010 (*n* = 1026)	2016 (*n* = 974)	*P*-value	2010 (*n* = 823)	2016 (n = 823)	*P*-value
Gender						
Male	437 (42.6)	333 (34.2)	< 0.001	339 (41.2)	311 (37.8)	0.158
Female	589 (57.4)	641 (65.8)		484 (58.8)	512 (62.2)	
Age						
70–74	317 (31)	293 (30.1)	0.108	259 (31.5)	248 (30.1)	0.923
75–79	319 (31.2)	264 (27.1)		255 (31)	254 (30.9)	
80–84	249 (24.3)	270 (27.7)		200 (24.3)	209 (25.4)	
≥ 85	138 (13.5)	147 (15.1)		109 (13.2)	112 (13.6)	
Living districts						
HK Islands	210 (20.5)	292 (30.1)	< 0.001	196 (23.8)	229 (27.8)	0.154
Kowloon	310 (30.2)	357 (36.8)		302 (36.7)	277 (33.7)	
New Territories	505 (49.3)	320 (33)		325 (39.5)	317 (38.5)	
Social Security Assistance						
No	924 (90.1)	863 (88.6)	0.292	743 (90.3)	731 (88.8)	0.334
Yes	102 (9.9)	111 (11.4)		80 (9.7)	92 (11.2)	
Medical insurance coverage						
No	944 (93.5)	913 (95.4)	0.062	769 (93.4)	780 (94.8)	0.250
Yes	66 (6.5)	44 (4.6)		54 (6.6)	43 (5.2)	
Living status						
Living with others	799 (78.3)	749 (76.9)	0.467	637 (77.4)	614 (74.6)	0.184
Living alone	222 (21.7)	225 (23.1)		186 (22.6)	209 (25.4)	

Figures may not add up to total number of respondents because of missing data

### Awareness of voucher scheme

The majority of respondents (99.3%) were aware of the Elderly Healthcare Voucher Scheme in 2016, a significant increase from 69.7% in 2010. The most common channel for knowing about the scheme was television advertisements (42.2%).

### Attitudes towards the voucher scheme

#### Design of the voucher scheme

Generally, the elderly were more positive about the design of the voucher scheme when compared with the findings in 2010 (Table 2). The majority felt the amount of information about the voucher scheme was fair or sufficient in 2016 (82.3%), a significant increase from 58.9% in 2010. Currently, 94.9% agreed that the voucher is convenient to use, as compared to 75.7% in 2010. 81.9% agreed that coverage of services was sufficient, an increase from 58.2% in 2010. Slightly less than half (49.5%) thought the current annual voucher amount (HK$2000 per year) is sufficient. However, the agreement was significantly higher than 20.2% in 2010 when the voucher amount was HK$250 per year.

**Table 2:** Attitudes toward voucher scheme among the matched respondents in 2010 and 2016

Variable	2010 (*N* = 823) *n (%)*	2016 (*N* = 823) *n (%)*	*P*-value
Whether sufficient information provided on the voucher scheme *(among those who were aware of the voucher scheme)*			
Sufficient	129 (27.9)	397 (53.1)	< 0.001
Fair	143 (31.0)	218 (29.2)	
Insufficient	190 (41.1)	132 (17.7)	
Whether voucher is convenient to use			
No	168 (24.3)	41 (5.1)	< 0.001
Yes	523 (75.7)	758 (94.9)	
Whether the service coverage of voucher is enough			
No	218 (41.8)	133 (18.1)	< 0.001
Yes	304 (58.2)	600 (81.9)	
Whether the subsidy amount of voucher is enough			
No	562 (79.8)	374 (50.5)	< 0.001
Yes	142 (20.2)	367 (49.5)	
Whether the voucher can encourage the use of health services in private sector			
No	468 (63.8)	305 (38.5)	< 0.001
Yes	266 (36.2)	488 (61.5)	

Figures may not add up to total number of respondents since we exclude those saying “don’t know”/"don’t remember” or missing;

*P*-value indicates the statistical significance of years with each variable

#### Perceived change of health seeking behavior

61.5% reported that “the scheme can encourage me to use more private primary care services”, which was an increase from 36.2% in 2010. Among those who agreed in 2016, the majority thought the voucher scheme would encourage them to use acute services in the private sector (90.3%), rather than preventive care (40.3%) and chronic disease management (12.2%). For those who disagreed, the common reasons for not changing their behavior included “It depends on what kind of illness e.g. the treatment of chronic disease will remain in the public system” (29.0%) and “I am used to seeing doctors in the public system” (27.4%).

#### Predictors on perceived change of health seeking behavior

Univariate analyses adjusted by year and enumeration sites showed that age, living district, living status, presence of social security assistance, health insurance coverage and usual source of care prior to the adoption of voucher were statistically significant with respect to whether the voucher would encourage the use of private primary care services. After putting all significant variables into the multivariate logistic regression model, the success of vouchers in encouraging the use of private healthcare services related to a number of factors including gender, age, and socioeconomic status. The elderly people who found the voucher scheme encourages them to use private primary care services were those who are used to “seeing both public and private doctors” prior to the adoption of voucher (OR: 1.28; CI: 0.98–1.67) than those “seeing only public doctors”, and those who lived in a district with lower median monthly domestic household income i.e. in Kowloon District (OR: 1.80; CI: 1.34–2.42) relative to those living in Hong Kong Islands (which has the highest income level). On the other hand, those elderly with health insurance (OR: 0.45; CI: 0.27–0.77), being female (OR: 0.78; CI: 0.61–0.98) and those who were older than 85 years old (OR: 0.59; CI: 0.40–0.85) relative to those aged 70–74, were less likely to consider vouchers to encourage them to use private primary care services (Table 3).

**Table 3:** Univariate and multivariate analysis of “Whether the voucher can encourage the use of health services in private sector”

				Univariate Analysis		Multivariate Analysis	
Variable	Total	No (*n* = 773)	Yes (*n* = 754)	Adjusted Odds ratio (95% CI)	*P*-value	Adjusted Odds ratio (95% CI)	*P*-value
*Demographic*							
Sex							
Male	650 (39.5)	295 (38.2)	316 (41.9)	1		1	
Female	996 (60.5)	478 (61.8)	438 (58.1)	0.81 (0.65–1.00)	0.052	0.78 (0.61–0.98)	0.032
Age							
70–74	507 (30.8)	225 (29.1)	248 (32.9)	1		1	
75–79	509 (30.9)	239 (30.9)	242 (32.1)	0.92 (0.70–1.19)	0.514	0.94 (0.71–1.25)	0.667
80–84	409 (24.8)	196 (25.4)	180 (23.9)	0.80 (0.60–1.06)	0.117	0.81 (0.60–1.10)	0.180
≥ 85	221 (13.4)	113 (14.6)	84 (11.1)	0.62 (0.43–0.88)	0.007	0.59 (0.40–0.85)	0.005
Living districts							
HK Islands	425 (25.8)	231 (29.9)	164 (21.8)	1		1	
Kowloon	579 (35.2)	249 (32.2)	302 (40.1)	1.92 (1.46–2.54)	< 0.001	1.80 (1.34–2.42)	< 0.001
New Territories	642 (39)	293 (37.9)	288 (38.2)	1.32 (1.00–1.75)	0.052	1.30 (0.96–1.76)	0.094
Received social security assistance							
No	1474 (89.6)	708 (91.6)	657 (87.1)	1		1	
Yes	172 (10.4)	65 (8.4)	97 (12.9)	1.55 (1.10–2.20)	0.013	1.36 (0.93–2.01)	0.114
Had health insurance coverage							
No	1549 (94.1)	714 (92.4)	724 (96)	1		1	
Yes	97 (5.9)	59 (7.6)	30 (4.0)	0.54 (0.34–0.87)	0.011	0.45 (0.27–0.77)	0.003
Living status							
Living with others	1251 (76)	610 (78.9)	557 (73.9)	1		–	
Living alone	395 (24)	163 (21.1)	197 (26.1)	1.31 (1.02–1.68)	0.036	1.25 (0.94–1.66)	0.121
*Health condition*							
Doctor consultation in past one month							
No	479 (29.2)	210 (27.4)	233 (30.9)	1		–	
Yes	1160 (70.8)	557 (72.6)	520 (69.1)	0.93 (0.72–1.20)	0.583		
Hospitalization in the past one year							
No	1290 (78.9)	606 (78.8)	590 (78.9)	1		–	
Yes	344 (21.1)	163 (21.2)	158 (21.1)	0.90 (0.70–1.17)	0.436		
Health condition when compared with other people at the same age							
Better	611 (39.1)	292 (39.9)	282 (39.4)	1		–	
Fair	736 (47.1)	350 (47.8)	325 (45.5)	0.94 (0.74–1.18)	0.586		
Worse	216 (13.8)	90 (12.3)	108 (15.1)	1.09 (0.78–1.54)	0.603		
Had chronic disease							
No	327 (20)	160 (20.8)	139 (18.5)	1		–	
Yes	1310 (80)	608 (79.2)	612 (81.5)	0.95 (0.72–1.24)	0.684		
Usual source of care (prior to the use of vouchers)							
Public doctors only	446 (29.3)	238 (32.6)	185 (26.9)	1		1	
Private doctors only	332 (21.8)	173 (23.7)	130 (18.9)	0.95 (0.69–1.3)	0.752	0.95 (0.69–1.32)	0.778
Both public and private doctors	743 (48.8)	320 (43.8)	373 (54.2)	1.31 (1.01–1.7)	0.041	1.28 (0.98–1.67)	0.069

Both analyses were adjusted by year and recruitment site

Figures may not add up to total number of respondents since we exclude those saying “don’t know”/"don’t remember” or missing;

*P*-value in univariate analysis indicates the statistical significance of the outcome measure i.e. whether to encourage private health services use with each independent variable

Only significant variables (except sex and age) in univariate analysis were put into multivariate analysis

### Vouchers usage

There was a marked increase in the number of matched elderly who had used voucher scheme, increasing to 94.7% from 36.4% in 2010 (Table 4). Voucher users are more likely to be older. Among those who have ever used vouchers, most used vouchers for acute services (90.2%) (from Department of Health’s data) compared to 82.0% in 2010. In 2016, only 29.7% used vouchers for preventive services, an increase from 7.4% in 2010. The percentage of those using vouchers for chronic disease management was similar in 2010 (7.8%) and 2016 (9.1%). Among those who had not used vouchers for preventive care and chronic diseases in 2016, 56.8 and 15.6% would consider using voucher for these services respectively.

**Table 4:** Voucher usage among the matched respondents in 2010 and 2016

Variable	2010 (*n* = 823)	2016 (*n* = 823)	*P*-value
Ever use			
No	497 (63.6)	43 (5.3)	< 0.001
Yes	284 (36.4)	773 (94.7)	
*Among those who have ever used the vouchers*			
For acute services*			
No	51 (18.0)	65 (9.8)	< 0.001
Yes	232 (82.0)	601 (90.2)	
For preventive services			
No	262 (92.6)	538 (70.3)	< 0.001
Yes	21 (7.4)	227 (29.7)	
For chronic disease management			
No	261 (92.2)	690 (90.9)	0.503
Yes	22 (7.8)	69 (9.1)	

* Result of 2016 is calculated from Department of Health 2009–2015 data

Figures may not add up to total number of respondents since we exclude those saying “don’t know”/"don’t remember” or missing;

*P*-value indicates the statistical significance of years with each variable

When asked how elderly perceived their choice of seeing public and private doctors in 2016 and prior to their use of voucher (Table 5), 61.9% said their current usual choice of care was “visiting both public and private doctors” which was a significant increase from 48.4% prior to their use of voucher. The percentage of “visiting only public doctors” and “visiting only private doctors” as their current usual choice of care was 17.2 and 17.4% respectively in 2016, which was a decrease from 24.7 and 20.8% prior to their use of voucher respectively. The percentage choosing public doctors as their current usual source of care i.e. combining respondents choosing “only public doctors” and “both public and private doctors” was 79.1% in 2016, an increase from 73.1% prior to their use of vouchers.

**Table 5:** Perceived usual source of care currently and prior to the use of voucher (2016)

Usual source of care	Prior to the use of vouchers (*n* = 766)	Currently (*n* = 766)	*P*-value
Public doctors only	189 (24.7)	132 (17.2)	< 0.001
Private doctors only	159 (20.8)	133 (17.4)	
Both public and private doctors	371 (48.4)	474 (61.9)	
Seldom/never seen doctors	44 (5.7)	26 (3.4)	
Seeing other healthcare professionals	3 (0.4)	1 (0.1)	

Figures may not add up to total number of respondents since we exclude those saying “don’t know”/"don’t remember” or missing;

*P*-value indicates the statistical significance of years with usual source of care

## Discussion

This repeated cross-sectional study shows that the current voucher scheme is more acceptable to the elderly than at the time of launch in 2009, reflected by the increased awareness and positive attitudes towards the design of the voucher, as compared with the survey conducted in 2010. The voucher seems to have encouraged elderly people to use primary care services available in the private sector, in particular for one-off (episodic) curative services rather than for preventive services or chronic disease management. There also appears to be an increase in the dual utilization of public and private sectors, with greater numbers choosing both sectors as their usual source of care. This was not the intention at the introduction of the voucher scheme.

### Impact of voucher scheme on services utilization

Vouchers are a demand-side subsidy intended to reduce financial hardship when accessing services and to improve efficiency [16]. Vouchers are also a useful means of targeting specific populations, and can improve the quality of services through incentivizing behavior change on both demand and supply side [11–13]. Evidence of effectiveness of vouchers has been demonstrated in encouraging people to perform clearly defined, time-limited and simple behavioral tasks [11–13, 19, 35]. However, there is insufficient evidence to demonstrate the effectiveness of vouchers for other applications. In its current design, the voucher scheme in Hong Kong provides a universal voucher, introduced to incentivize the entire Hong Kong resident population aged 65 and above to choose ten different types of primary care practitioners for curative care, preventive care and chronic diseases management in the private sector. The elderly particularly those with chronic disease in Hong Kong frequently use the highly subsidized healthcare services in public sector, mainly due to lower user fees in public sector and lower trust in the private sector [28]. Our results show the elderly perceive that the voucher has encouraged them to use private primary care services over the past few years (an increase from 36.2% in 2010 to 61.5% in 2016), probably due to the effect of an eightfold-increase in the subsidy amount. Those elderly who are less affluent are more likely to see the vouchers as encouraging them to use private primary care services. Vouchers also encourage those elderly who are used to seek services in both the public and private sectors (relative to those “only seeing public doctors”) to use private primary care services. The effect on encouraging private primary care on patients who used to “seeing only public doctors” was less evident. This may be due either to the preference and trust in the public sector or to the need for chronic disease management which may be unaffordable even with the voucher, to low income elderly persons.

In addition, the percentage of dual utilization of both public and private sectors as their usual source of care is increasing, suggesting that the vouchers do not have a substitution effect of private services on public services utilization to reduce the burden of care on public sector. This may also be undesirable as it compromises continuity of care, and encourages “doctor shopping” which a phenomenon already observed and may affect the quality of care.

### Impact of voucher scheme on primary care and disease prevention

It appears the elderly are less likely to be incentivized to use private primary care services for prevention or management of chronic disease due to the untargeted nature of voucher scheme in Hong Kong. This finding suggests that greater attention is needed in promoting the use of vouchers to achieve program objectives and increase their effectiveness. The use of vouchers for preventive services such as health and dental checks has remained low over the past 8 years of voucher implementation. This could be due to low awareness of preventive care among elderly people, but also to the untargeted nature of the voucher design which covers a spectrum of health services. Preventive care is an important component of primary care for disease prevention and maintenance within a community setting [1]. The literature suggests that vouchers can be effective in encouraging uptake of well-defined and time-limited services that are under-utilized. A designated voucher for preventive care services for the elderly should be instituted to increase uptake for disease prevention. This approach might encourage the elderly to take greater precautionary measures against diseases, enhance their capability in self-care, and reduce the demand for hospitalisation. It can also save medical costs in the long run by screening out the high risk group for early treatment. Since the need for chronic disease management has not been met which may be related to affordability, a designated voucher for chronic disease management needs to be considered for those who are screened to have chronic disease. Looking to the future, the increasingly ageing population and growing chronic disease burden in Hong Kong will require a greater emphasis on integrated community health services to reduce demand on the hospital sector.

### Implementation issues

From the supply side perspective, vouchers can be perceived as a policy to address health service gaps and engaging private service providers [36]. The prices of goods and services are reduced through competition between providers. In Hong Kong, the participation rate of primary care practitioners is relatively low at 20% in 2017 [32]. Some healthcare providers have not enrolled because of the associated administrative burden [26]. Though enrolment has been gradually increasing, access for elderly people wishing to use vouchers is suboptimal. Increasing the number and choices of providers could facilitate greater uptake. Another important aspect of the voucher scheme is the indirect process of regulating providers, using contracting to promote minimum standards of quality as a prerequisite to enrolling in the voucher scheme, thereby increasing the quality of service provision [36]. A few studies in a systematic review demonstrated that engaging the private sector in providing services for low income groups might increase quality and utilization [37]. Clearly defined and shared objectives and roles, transparency of information, and trust are key elements in building partnerships with the private sector [9]. Active involvement of the private sector in designing the voucher scheme could facilitate more utilization of specific health services though incentivizing both the demand and supply sides.

A well-implemented voucher program should bring benefit to both key stakeholders of the recipients and service providers, and maximize service delivery capacity as a whole for equitable access in universal health coverage and contribute to a sustainable financing system. As a demand-side financing mechanism, it enables the government to funnel resources to population in need. It can reduce barrier in access to health services and extend private sector’s reach by expanding the population purchasing power through subsidy. The design of vouchers, including lowering the eligible age for participation in the scheme to promote uptake of preventive services and control of non-communicable diseases, and specification of the type of preventive care and chronic diseases management programs to be targeted, needs to be urgently considered and investigated. Vouchers need to be introduced in a systematic manner and to be targeted at interventions which can be evaluated to ensure value for money, quality and effectiveness. The effectiveness of vouchers will rely on engaging fully with services providers as well as targeting specific populations, and simple administrative processes will be needed for effective implementation [38].

There were a few limitations in this study. Due to the lack of a population register from which the elderly can be randomly sampled in Hong Kong, a convenience sample recruiting elderly people from clinics, health centres, elderly centres/ parks from different districts in Hong Kong was used to reflect different socio-economic characteristics. To assess the representativeness of our sample, we compared the age and sex distribution of respondents with the population in each year, and the data did not differ significantly. We have also used propensity score matching so that the differentials in demographic and socio-economic factors between years can be properly adjusted. On the other hand, as our results were based on cross-sectional surveys, the associations between perceived change of health seeking behavior and other variables do not imply causality, which require confirmation in longitudinal studies. Finally, there is limitation in the use of self-reported measures, such as the elderly’s use of vouchers and their health status. The administrative data from the Department of Health regarding the voucher usage has been used to validate the self-reported voucher usage by the respondents.

## Conclusions

Vouchers can increase service utilization for a particular service and can leverage private sector provision to fill gaps in public provision by targeting a particular group. However, in Hong Kong the voucher system for the elderly population has not enhanced the utilization of primary care for prevention and maintenance of chronic conditions within a community setting due to its unspecified design. The voucher scheme also appears to increase the dual utilization of health services in both the public and private sectors which may compromise continuity and quality of care. The voucher scheme has encouraged the use of healthcare services in the private sector, however, there needs to be caution that the untargeted and open-ended nature of voucher scheme could result in supply-induced demand which would affect long term financial sustainability. The voucher needs to be re-designed and be more specific about the range of services covered and how they are funded, managed, and delivered to target prevention and chronic diseases management rather than unspecified care which is mainly acute and episodic in order to meet the needs of the elderly population. Apart from administrative enhancement and technical changes in the subsidy amount and eligible age, a change in the targeting and scope of services is required to effectively achieve the intended goals to maximize service delivery capacity as a whole for equitable access in universal health coverage and to contribute to a sustainable financing system.

## Supplementary information

**Additional file 1.** Questionnaire.

## Data Availability

The datasets analyzed during the current study are not publicly available due to the funding requirement, but are available from the corresponding author on reasonable request.
