# Supplementary material for: What is the long term impact of voucher scheme on primary care? Findings from a repeated cross sectional study using propensity score matching
Source: BMC Health Serv Res. 2019 Nov 21;19:875. doi: 10.1186/s12913-019-4707-8 (PMC6873583; doi:10.1186/s12913-019-4707-8)
Supplement: Supplementary file 1 — Additional file 1. Questionnaire. [file 12913_2019_4707_MOESM1_ESM.docx]

**Additional File 1 - Questionnaire**

**SCREENING QUESTION**

1. **Are you Hong Kong resident?**
2. Yes
3. No (End the interview)
4. **Are you aged 70 or above?**
5. Yes; Your Age: 1. 70-74 2. 75-79 3. 80-84 4. 85-89 5. ≥90
6. No (End the interview)

**(A) Awareness of Elderly Health Care Voucher Scheme**

1. **Are you aware of the Elderly Health Care Voucher Scheme?*(Please present the cue card with Scheme logo to respondents to facilitate their recalling of his/her awareness)***
2. Yes
3. No (Skip to “Section D: Attitude towards the Voucher Scheme”)

- Explain the followings:

***(Please present the cue card and explain the Scheme information to respondents)***

*“All elderly aged 70 or above, holding a valid HK Identity Card or Certificate of Exemption will be provided with vouchers annually as partial subsidy for using private primary care services, including the curative, preventive and rehabilitative care provided by ten types of healthcare professionals. Currently, the annual voucher amount is $2,000. You do not need to pre-register, collect or carry the voucher. To use vouchers, you just need to visit a healthcare professional who is enrolled under the Voucher Scheme and show the identity document required. Currently, over 5,200 healthcare professionals practicing in over 8,000 practices are enrolled under the Voucher Scheme”*

1. **How do you know about the Voucher Scheme? Anymore? *(Please check all that apply and present to respondents the publicity materials for eas-y reference)***

|  | 1. Press/ Magazines |
| --- | --- |
|  | 1. Posters/ leaflets |
|  | 1. Internet |
|  | 1. Words of mouth from family/ relatives/ friends |
|  | 1. Elderly centres (e.g. District Elderly Community Centre/ Neighbourhood Elderly Centre/ Social centres for the Elderly ) / health talks/ social workers |
|  | 1. Radio advertisements |
|  | 1. Television advertisements |
|  | 1. Healthcare professionals/ clinics of private sector and public sector (e.g. outpatient clinics of Hospital Authority) |
|  | 1. Others, please specify: _________________ |

1. **Do you know which types of healthcare professionals could join the current Voucher Scheme?**
2. Yes
3. No (Skip to Q7)

*(For those who know which healthcare professionals could join)*

1. **Please tell me three types of healthcare professionals who you think are eligible to join the current Voucher Scheme. Anymore? *(Please circle the first three types of healthcare professionals named by the elderly and tick the other healthcare professionals named by the elderly in the table below.)***

|  | 1. Registered medical doctors |  | 1. Registered Chinese medicine practitioners |
| --- | --- | --- | --- |
|  | 1. Registered dentists |  | 1. Registered chiropractors |
|  | 1. Registered nurses, enrolled nurses |  | 1. Registered physiotherapists |
|  | 1. Registered occupational therapists |  | 1. Registered radiographers |
|  | 1. Registered medical laboratory technologists |  | 1. Optometrists with part 1 registration |
|  | 1. Others, please specify:_________________________________________   *(Please note that other healthcare professionals mentioned by the elderly are incorrect answers.)* | | |

1. **Do you think the information about the Voucher Scheme provided to you is sufficient or not?**
2. Sufficient (Skip to Q9)
3. Fair (Skip to Q9)
4. Not sufficient
5. Don’t know (Skip to Q9)

*(For those who think the information is "Not sufficient")*

1. **What kind of information would you like to enrich? Anymore? *(please check all that apply)***

|  | 1. Channels to disseminate the list of enrolled healthcare service providers |
| --- | --- |
|  | 1. How to use the voucher |
|  | 1. How to check my voucher account balance |
|  | 1. Others, please specify: _________________ |

**Read out to all respondents:**

*Elderly Health Care Voucher Scheme was launched in 2009. All elderly aged 70 or above, holding a valid HK Identity Cared or Certificate of Exemption, will be given a fixed amount of subsidy per year to use private primary care services. The amount of vouchers given per year was $250 in 2009. Since 2012, Government has many enhancements on the Voucher Scheme****.***

**(B) Understanding of the Voucher Scheme**

**We would like to explore how much you know about these changes on Voucher Scheme. *[For Interviewers, please inform the respondents of the correct answer if the respondents do not know or give an incorrect answer for Q9 to Q16.]***

1. **Do you know the current amount of voucher for each elderly every year? [Correct answer is $2,000]**
2. Yes→ **What is the current amount of voucher per year now?** HK$_______
3. No
4. **Unspent vouchers of an eligible elderly can be carried forward for his use in subsequent years. Do you know whether there is a ceiling of the accumulated vouchers in the voucher account? [Correct answer is $4,000]**
5. Yes→ **What is the current accumulation limit?** HK$_______
6. No
7. **From 2009 to 2011, 5 vouchers of $50 each were provided to the eligible elderly annually. For example, if the consultation costs HK$230, you need to use 4 vouchers (HK$200), and pay the extra HK$30 with cash. Do you know there is a change in the face value of each voucher? [Correct answer is $50🡪$1] (pilot test)**
8. Yes → **What is the change in face value?** HK$_______
9. No
10. **Do you think the voucher now can be used in Mainland or not? [Correct answer is University of Hong Kong- Shenzhen Hospital]**
11. Yes → **Where can it be used?** _______________________
12. No
13. Don’t know
14. **Do you know how to check your voucher balance? [Correct answer is the Scheme website, through telephone (Interactive Voice Response System), by the voucher balance records after voucher use (“the Notice on Use of Health Care Voucher”) and from the attending healthcare professional]**
15. Yes → **Where?**
16. the Scheme website
17. By telephone
18. By “the Notice on Use of Health Care Voucher”
19. By attending healthcare professionals
20. Others___________________________
21. No
22. **Can you use vouchers to settle hospital in-patient charges? [Correct answer is NO]**
23. Yes
24. No
25. Don't know
26. **Can you use vouchers to buy herbal medicines/ medication / medical equipment without healthcare professional’s consultations？ [Correct answer is NO]**
27. Yes
28. No
29. Don't know
30. **Can you use vouchers to buy medicines for your family? [Correct answer is NO]**
31. Yes
32. No
33. Don't know
34. **Have you ever used the voucher?**

**(C) Vouchers usage**

1. Yes (Skip to Q19)
2. No
3. Don't know (Skip to "Section D: Attitude towards the Voucher Scheme")

*(For those who have never used vouchers)*

1. **Why have you never used vouchers? Anymore? *(Do not read out) (please check all that apply)***
2. I am not aware of the Voucher Scheme [Interviewer, check with Q3 for consistency]
3. Too little amount
4. Could not find an enrolled healthcare professional nearby
5. The private healthcare professional whom I usually saw has not enrolled in the Voucher Scheme
6. Complicated procedure in using the voucher
7. I seldom consult doctors/ other healthcare professionals
8. I prefer using public healthcare services e.g. Government, Hospital Authority’s services
9. Others, please specify:__________________________

(Then Skip to "Section D: Attitude towards the Voucher Scheme")

*(For those who have used vouchers)*

1. **Have you encountered any difficulties in locating the enrolled healthcare professionals you need?**
   - - 1. Yes – 19a. **What are the difficulties?** **Anymore? *(Do not read out) (please check all that apply)***
2. Don't known which service providers are registered in the Voucher Scheme
3. Not all private healthcare professionals have joined the Voucher Scheme
4. The private healthcare professional whom I usually saw is not enrolled in the Voucher Scheme
5. Others; please specify: ________
   - - 1. No
6. **How do you know which healthcare professionals have joined the Voucher Scheme? Anymore? *(Do not read out) (Please check all that apply)***

|  | 1. Looking at the Scheme logo at their location of practice |
| --- | --- |
|  | 1. Going to internet to check the lists of enrolled healthcare professionals |
|  | 1. Words of mouth from family/ relatives/ friends |
|  | 1. Enquiry hotline of Department of Health/ the Voucher Scheme |
|  | 1. Searching conducted by relatives/ family members |
|  | 1. I do not need to search for the list. Please specify the reason _________________________________________ |
|  | 1. Others, please specify: ___________________ |

1. **What are the reasons for you to use the voucher? Anymore? *(Do not read out) (Please check all that apply)***

|  | 1. Making good use of the subsidy |  | 1. To save money |
| --- | --- | --- | --- |
|  | 1. Shorter waiting time in seeing private healthcare professionals |  | 1. Cannot make appointment in public healthcare services |
|  | 1. Better quality in private sectors |  | 1. Healthcare professionals/ clinic staff suggest using the vouchers |
|  | 1. Healthcare professionals/ clinic staff suggest using the vouchers |  | 1. Others, please specify: _________________________ |

1. **The government encourages the public to use vouchers for preventive care such as health check, vaccination or dental check-up. Did you use vouchers for these preventive care?**
   - - 1. Yes
       2. No → 22a. **Will you consider using vouchers for preventive care?**
2. Yes
3. No, please state reasons ___________________________
4. Don’t know
   - - 1. Don’t know
5. **Will you plan to use voucher for chronic disease management such as hypertension, diabetes?**
   - - 1. Yes
       2. No → 23a. **Will you consider using vouchers for chronic disease management?**
6. Yes
7. No, please state reasons ___________________________
8. Don’t know
   - - 1. Don’t know
9. **Before using the voucher, did you usually consult public doctors or private doctors or both?**
10. Public doctors

24a. **At that time, did you used to consult a consistent private western medical doctor frequently?** “A consistent and frequently visited doctor” refers to the doctor you will go to when you are sick (including cold and flu) or needed preventive healthcare service.

1. Yes
2. No
3. Don’t know

1. Private doctors
2. Both
3. Seldom/ never seen doctors
4. Others:____________
5. **Currently, do you usually consult public doctors or private doctors or both?**
6. Public doctors

25a. **After using the vouchers, have you been consulting a consistent private western medical doctor frequently?** “A consistent and frequently visited doctor” refers to the doctor you will go to when you are sick (including cold and flu) or needed preventive healthcare service.

1. Yes
2. No
3. Don’t know
4. Private doctors
5. Both
6. Seldom/ never seen doctors
7. Others:_______________

(For interviewer, for questions using 5-point likert scale on agreement, please firstly (1) ask respondent if they agree or not; (2) if respondents answer "agree", probe whether they are "agree" or "strongly disagree"; if respondents answer "disagree", probe whether they are "disagree" or "strongly disagree") *show the cue card*

**(D) Attitudes towards the Voucher Scheme**

1. **Do you agree “Voucher is convenient to use”?**
2. Strongly agree (Go to Q 28)
3. Agree (Go to Q 28)
4. Disagree
5. Strongly disagree
6. Don’t know (Go to Q 28)

*(For those who disagree i.e. not convenient)*

1. **How to make it more convenient to use? Anymore? *(Do not read out)* (*Please check all that apply*)**

|  | 1. Use paper vouchers |
| --- | --- |
|  | 1. More publicity on how to use it |
|  | 1. Make it compulsory for all healthcare professionals to join |
|  | 1. Others, please specify: ___________________ |

1. **With the current annual voucher amount of $2,000 per eligible elderly, do you agree that “the annual voucher amount is just about right”?**
2. Yes, just about right (Go to Q30)
3. No, too low
4. No, too high
5. Don’t know (Go to Q30)

*(For those who think the amount is too low or too high)*

1. **How much would be reasonable?**

$ ______

1. **Do you agree that “current level of the accumulation limit of $4,000 is just about right”?**
2. Yes, just about right (Go to Q32)
3. No, too low
4. No, too high
5. Don’t know (Go toQ32)

*(For those who think the accumulation limit is too low or too high)*

1. **How much would be reasonable?**

$ ______

1. **Do you think the Voucher Scheme will encourage you to use private primary care services?**
2. Strongly agree
3. Agree
4. Disagree (Go to Q34)
5. Strongly disagree (Go to Q34)
6. Don’t know (Go to Q35)

*(For those who “agree” or “strongly agree” i.e. the Voucher Scheme has encouraged the use of private primary care)*

1. **In what ways would the Voucher Scheme encourage you to use the vouchers for private primary care services? *(Please read out the following options one by one and check each option by yes, no or don't know) Anymore?***

|  | Yes | No | Don’t know | NA |
| --- | --- | --- | --- | --- |
| - - - 1. Seek healthcare services in the private sector more often (e.g. cold and flu) |  |  |  |  |
| - - - 1. Use more preventative care (e.g. dental check-up, vaccination, body check) |  |  |  |  |
| - - - 1. Use more chronic illness management services |  |  |  |  |
| - - - 1. Receive more healthcare services provided by different types of healthcare professionals (e.g. dental, optometry etc) |  |  |  |  |
| - - - 1. See a (consistent) family doctor more often |  |  |  |  |
| - - - 1. Others, please specify: _________________ |  |  |  |  |

*(For those who “disagree” or “strongly disagree” i.e. the Voucher Scheme has not encouraged the use of vouchers for private primary care services)*

1. **Can you tell me why do you think that the Voucher Scheme is not useful in encouraging you to seek primary care services? Anymore?  *(Do not read out) (Please check all that apply)***

|  | 1. Too little amount |
| --- | --- |
|  | 1. Complicated procedure in using the voucher |
|  | 1. Not all private healthcare professionals join the Voucher Scheme |
|  | 1. The private healthcare professionals I usually visit have not enrolled in the Voucher Scheme |
|  | 1. I prefer using/am only using public healthcare services e.g. Gov’t, HA services |
|  | 1. Depends on what kind of illness I have, if it is chronic illness I’d prefer to stay in the public healthcare system |
|  | 1. I do not need to use vouchers |
|  | 1. Others, please specify: _________________ |
|  | 1. Don’t know |

1. **Do you agree “The coverage of the types of healthcare services under the Voucher Scheme is sufficient”?**
2. Strongly agree (Go to Q 37)
3. Agree (Go to Q37)
4. Disagree
5. Strongly disagree
6. Don’t know (Go to Q 37)

*(For those “disagree” or “strongly disagree” i.e. coverage not enough)*

1. **What items do you want to add in the service scope of the Voucher Scheme? (Please clarify if the item raised has already included in the Scheme) *(Do not read out) (Please check all that apply)***

|  | 1. Allied health professionals (e.g. Clinical Psychologists) |
| --- | --- |
|  | 1. Hospitalization |
|  | 1. Purchase herbal medicine, medication and medical equipment without consultations |
|  | 1. Others, please specify: |

1. **[Only for those have used vouchers] For the same type of medical services provided by same healthcare professional, did the healthcare professional charge you more when vouchers are used?**
2. Yes 🡪 Most of the consultations
3. Yes 🡪 Some of the consultations
4. Yes 🡪 Rarely
5. Never
6. Don't know
7. **In order to prevent over-charging for unnecessary services of the voucher users, do you agree to set an upper limit for each consultation?**
8. Strongly agree
9. Agree
10. Disagree
11. Strongly disagree
12. Don't know
13. **Do you agree that a portion of the voucher to be preserved for preventive healthcare services only, for example health check, vaccination, dental check-ups , and not for other usages, for example general consultations or other dental services?**
14. Strongly agree
15. Agree
16. Disagree
17. Strongly disagree
18. Don't know

**(E1) Respondent’s profile (Demography & socioeconomic status)**

1. **Your Gender:**
2. Male
3. Female
4. **What is your current living status?**
5. Live alone
6. Live with family / others: _______________
7. Live in institution (Hospital /Convalescent Hospital / Rehabilitation Hospital)
8. Live in old age home
9. **Which district do you live?**

|  | 1. Central & Western |  | 1. Eastern |  | 1. Southern |
| --- | --- | --- | --- | --- | --- |
|  | 1. Wan Chai |  | 1. Kowloon City |  | 1. Kwun Tong |
|  | 1. Sham Shui Po |  | 1. Wong Tai Sin |  | 1. Yau Tsim Mong |
|  | 1. Islands |  | 1. Kwai Tsing |  | 1. North |
|  | 1. Sai Kung |  | 1. Shatin |  | 1. Tai Po |
|  | 1. Tsuen Wan |  | 1. Tuen Mun |  | 1. Yuen Long |

1. **Which of the following sources of income do you have/ or do you receive any kind of financial support? (Read out one by one) *(Please check all that apply)Any more?***

|  | 1. Job income/ Salary |
| --- | --- |
|  | 1. Pension |
|  | 1. Financial support from children/ other relatives |
|  | 1. Comprehensive Social Security Assistance Scheme (CSSA) |
|  | 1. Disability allowance |
|  | 1. Old Age Allowance |
|  | 1. Other sources of income (e.g. interests, bonus, rental income, etc.) |
|  | 1. No income |

1. **Including all sources of income and MPF contribution, how much is your monthly household income approximately? [pilot test]**
2. No income
3. Under HK$6,000
4. HK$6,000 to 11,999
5. HK$12,000 to $17,999
6. HK$18,000 to $23,999
7. HK$24,000 to $29,999
8. HK$30,000 to $39,999
9. HK$40,000 to $59,999
10. ≥HK$60,000
11. Not willing to answer/ Don’t know
12. **Do you have any health insurance coverage?**
13. Yes
14. No (Go to Q47)
15. Don’t know (Go to Q47)

*(For those with health insurance coverage)*

1. **What does it cover? Anymore? *(Do not read out) (Please check all that apply)***

|  | 1. Hospitalization |  | 1. General out-patient |
| --- | --- | --- | --- |
|  | 1. Specialist out-patient |  | 1. Dental consultation |
|  | 1. Chinese herbalist/acupuncturist/   bonesetter |  | 1. Annual medical check-up |
|  | 1. Provide a fixed amount of   reimbursement (not limited to specific item) |  | 1. Others, please specify:   _________________ |
|  | 1. Don’t know |  |  |

**(E2) Self-rated health and disease diagnosis**

1. **In the past 30 days, have you had a consultation with a doctor in Hong Kong, and how many times? Please include follow-up consultations, and exclude those for seeking medical advice by telephone or taking medicine only but without seeing the doctor. *(Multiple options allowed)***

|  | 1. No |
| --- | --- |
|  | 1. Yes, Private doctors of Western medicine in Hong Kong, ____________ times |
|  | 1. Yes, Public doctors in out-patient departments of hospitals under Hospital Authority _______________ times |
|  | 1. Yes, Public doctors in Accident & Emergency departments of hospitals under Hospital Authority _______________ times |
|  | 1. Yes, Public doctors in clinics /centres under Department of Health _______________ times |
|  | 1. Yes, Others (Please specify:_____________________ _______________ times |

1. **Have you been hospitalized in the past one year? *(Multiple options allowed)***

|  | 1. No |
| --- | --- |
|  | 1. Yes, in public hospital _______________ times |
|  | 1. Yes, in private hospital _______________ times |
|  | 1. Yes, Others (Please specify:_____________________ __________times |

1. **Comparing with other people of your same age, what do you think about your health condition?**
2. Much better
3. Better
4. Similar
5. Worse
6. Much worse
7. Don’t know
8. **Have you ever been told by a western doctor that you had the following chronic health conditions? *(Read out the answer one by one and then check all that apply)***

|  | 1. Diabetes mellitus |  | 1. High blood pressure |  | 1. Heart diseases |
| --- | --- | --- | --- | --- | --- |
|  | 1. Stroke |  | 1. Asthma |  | 1. High cholesterol |
|  | 1. Gout |  | 1. Cancer |  | 1. None of the above |
|  | 1. Don’t know |  | 1. Others, please specify: | | |

**End of the Questionnaire**

This is a longitudinal study. We wish to seek your opinion or follow-up with you again in near future. Might you leave your telephone number to us such that we could contact you later?

Telephone number: ___________________

In case if the questionnaire is completed by family members or/ relative of the elderly, please indicate:

Relationship of family members or/ relative with the elderly:

Reasons for family members or/ relative of the elderly to respond on behalf of the elderly

- - - 1. Dementia diagnosed by healthcare professional
      2. Hearing problems
      3. Language problems
      4. Mentally/ Cognitive incapacitated
      5. Others (Please specify ____________________________)

**Thank for your much for your participation in the Survey!**
